# Solar forcing of early Holocene droughts on the Yucatán peninsula

**DOI:** 10.1038/s41598-021-93417-z

**Published:** 2021-07-06

**Authors:** Sophie F. Warken, Nils Schorndorf, Wolfgang Stinnesbeck, Dominik Hennhoefer, Sarah R. Stinnesbeck, Julius Förstel, Simon D. Steidle, Jerónimo Avilés Olguin, Norbert Frank

**Affiliations:** 1 Institute of Environmental Physics, Heidelberg, Germany; 2 Institute of Earth Sciences, Heidelberg, Germany; 3 Department of Earth Sciences, Khalifa University, Abu Dhabi, UAE; 4 Institute of Earth Sciences, Natural History Museum, Karlsruhe, Germany; 5 Institute of Geology, University of Innsbruck, Innsbruck, Austria; 6 Museo del Desierto, Saltillo, Coahuila Mexico

**Keywords:** Hydrology, Palaeoclimate

## Abstract

A speleothem record from the north-eastern Yucatán peninsula (Mexico) provides new insights into the tropical hydro-climate of the Americas between 11,040 and 9520 a BP on up to sub-decadal scale. Despite the complex atmospheric reorganization during the end of the last deglaciation, the dominant internal leading modes of precipitation variability during the late Holocene were also active during the time of record. While multi-decadal variations were not persistent, Mesoamerican precipitation was dominated by changes on the decadal- and centennial scale, which may be attributed to ENSO activity driven by solar forcing. Freshwater fluxes from the remnant Laurentide ice sheet into the Gulf of Mexico and the North Atlantic have additionally modulated the regional evaporation/precipitation balance. In particular, this study underlines the importance of solar activity on tropical and subtropical climate variability through forcing of the tropical Pacific, providing a plausible scenario for observed recurrent droughts on the decadal scale throughout the Holocene.

## Introduction

The tropical hydrological cycle plays an important role in global climate modulation and in regulating modern rapid climate change^1–3^. Today, and for the most of the mid- to late Holocene, a significant decadal to centennial variability of precipitation is well attested in the tropical Atlantic region in accordance with Atlantic and Pacific SST variability, volcanic forcing and solar insolation changes^3–8^. Due to the regional distribution of land and the position close to both the tropical Atlantic and Pacific Oceans, the local response of the seasonal ITCZ and the associated trajectories and strength of the trade winds and convective precipitation patterns, is, however, regionally very complex and dynamic^9, 10^. The resulting environmental conditions on the Yucatán peninsula at the northernmost boundary of the influence of the ITCZ are characterized by prolonged inter-annual droughts, which for example have had severe consequences for the collapses of the mid- to late Holocene Maya empire^4, 5, 11^. Despite these unfavorable conditions, human presence on the northern Yucatán peninsula was attested dating back until the late Pleistocene [e.g.^12, 13^].

During the early Holocene, i.e. the period between 11,700 and 8200 a before present (BP = 1950), the terminal deglaciation followed upon the dramatic re-organization of global climate. During this epoch, northern hemisphere warming was almost completed, modern atmospheric and ocean circulation nearly established, and the melting of Laurentide and European ice sheets led to a sea-level of c. − 20 m below modern values^14^. Large meltwater pulses from the remnant Laurentide ice sheet into the North Atlantic (NA) ocean and the Gulf of Mexico yielded frequent lowering of sea surface temperatures (SST) on centennial to millennial timescales^15, 16^. These events coincided with a weak and southward shifted ITCZ, and, as a consequence, reduced precipitation in Central America and the northern Caribbean^16–20^. Nevertheless, the intensification of northern hemisphere summer insolation was accompanied by a progressive northward migration of the ITCZ, but the initiation of a stable convective hydro-climate in Central America occurred, although not before c. 9000 a BP^21^.

The atmospheric circulation over the Yucatán peninsula is shaped by the competition between the North Atlantic subtropical high pressure system and the eastern Pacific ITCZ, which influence the convergence patterns on seasonal and inter-annual timescales^22, 23^. This so-called Pacific-Atlantic ‘inter-basin mode’ is modulated by several forcing mechanisms, such as El Niño–Southern Oscillation (ENSO) variance, volcanic and solar radiative forcing, or variations in (sub-)tropical Atlantic SSTs^4, 6–8, 24^. However, over the modern era solar and volcanic forcing is masked by the increasing dominance of anthropogenic radiative forcing^25^. Over the course of the Holocene the evolution of the strength of ENSO variance was subject to pronounced changes^26^. During the mid-Holocene, ENSO was likely dampened and only increased to its present dynamic since c. 3000–4000 a BP^27–29^.

High-resolution paleoclimate information has been retrieved from e.g., lacustrine and speleothem-derived climate records spanning the mid- to late Holocene, which helped to understand how the global climatic system has reached its current state^5, 20, 21, 30^. In contrast, comparatively little is known about how early Holocene hydro-climate dynamics compared to the mid- and late Holocene, including periods and occurrence of prolonged droughts, the persistence of leading modes of climate variability (ENSO, AMO), or severe tropical cyclone activity. To fill the present knowledge gap regarding the early Holocene climate in Yucatán, and, in particular, to allow future research to put the numerous archaeological and paleontological findings into an environmental context, we here present a new multi-proxy record from a submerged stalagmite from the north-eastern Yucatán peninsula. This record provides evidence for pronounced precipitation variability and the occurrence of droughts on a decadal scale during the transition into the mid-Holocene, suggesting a climate similar to present-day.

**Figure 1:**
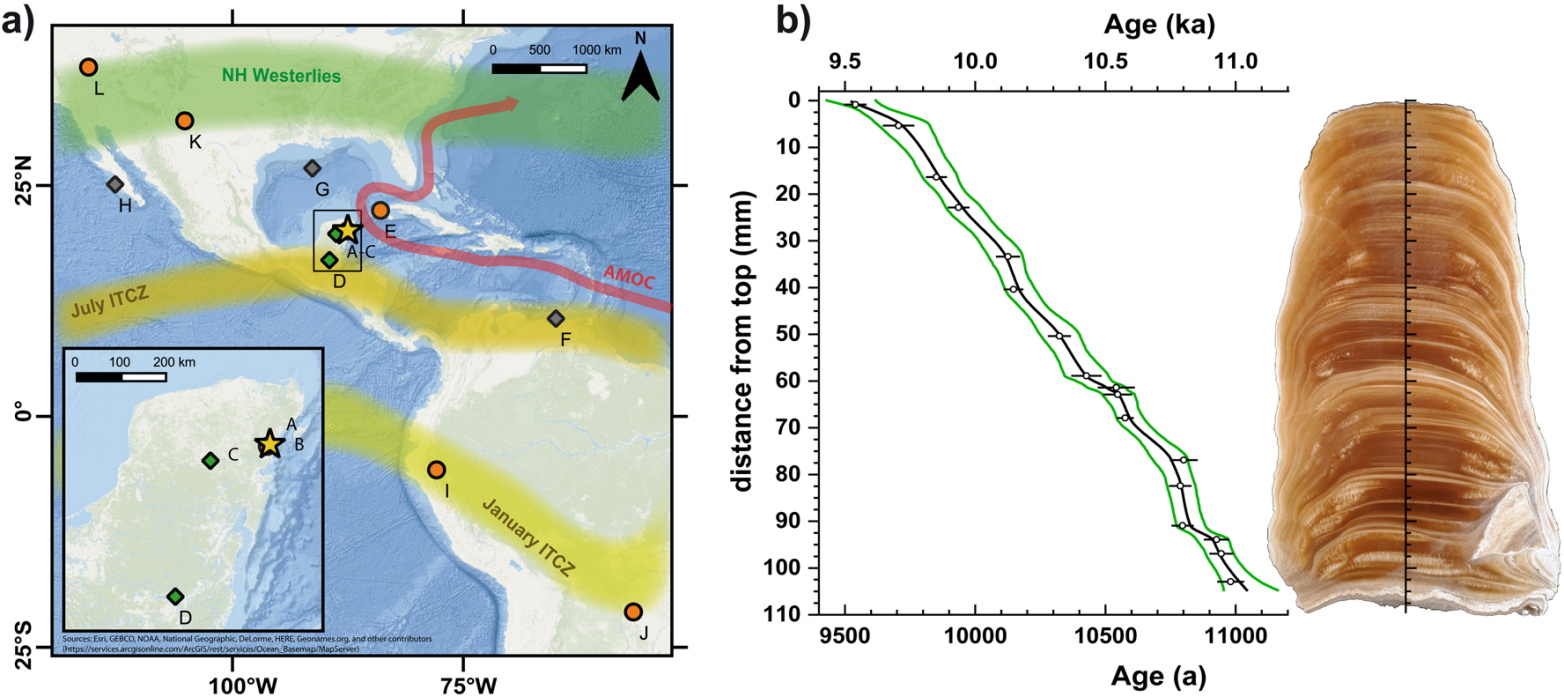
(**a**) Location of the Naharon sinkhole (A, yellow star) and positions of discussed studies. Orange circles indicate speleothem-based reconstructions, green diamonds lacustrine records, and gray diamonds marine sediment studies: (B) Chan Hol cave system^12^; (C) Lake Chichancanab^4, 11^; (D) Lake Petén Itzá^19^; (E) Dos Anas Cave System^20^; (F) Cariaco Basin^2^; (G) Gulf of Mexico^17, 31^; (H) Soledad basin (I) Shatuca cave, Peru^32^; (J) Jaraguá Cave, Brazil^33^ (K) Pink Panther Cave, New Mexico^34^; (L) Leviathan Cave^35^. The average annual northernmost (July) and southernmost (January) margins of the Intertropical Convergence Zone (ITCZ) as well as the region dominated by the NH westerlies are also shown (yellow and green bands, respectively). The climate in the region is further under direct influence of the strength of the surface ocean currents as a part of the Atlantic meridional overturning circulation (AMOC). Map created using the free and open source software QGIS v3.16.3 (http://www.qgis.org). (**b**) Age-depth-model of stalagmite NAH14 constructed with COPRA^36^. Green lines indicate the 2.5% and 97.5% confidence intervals, respectively. On the right a scan of NAH14 is shown. The stalagmite consists of translucent, honey-colored columnar calcite, frequently interrupted by white, opaque layers of sub-mm thickness.

Stalagmite NAH14 was collected by technical divers at a depth of − 17.7 m below sea level from the presently submerged Naharon sinkhole, which is part of the Naranjal cave system in the area of Tulum, Quintana Roo, Mexico (20° 11.93′ N, 87° 30.78′ W, Fig. 1). It covers the earliest phase of the Holocene on the north-eastern Yucatán peninsula. The speleothem constitutes an excellent archive to reconstruct past environmental conditions at unprecedented time resolution, and provides important new evidence on the geographical extent of centennial-scale climatic anomalies.

## Results

### Chronology

In total, 18 samples were analyzed for Th-U-dating from stalagmite NAH14 (Table S1, Fig. 1). The specimen has a moderately high U content ranging between 700 and 1600 ng g^-1^. The natural most abundant ^232^Th isotope has a low abundance of < 0.3 ng g^-1^, which results in (^230^Th/^232^Th) activity ratios of > 3000, indicating negligible residual detrital contamination. However, since elevated initial ^230^Th concentrations were reported for several caves on the Yucatán peninsula, we here use an initial (^230^Th/^232^Th) ratio of 3.5 ± 1.8 to correct activity ratios for initial ^230^Th and ages^37, 38^. The resulting age correction yields an average value of 10 a, which is within uncertainty of the analytical precision of the corrected ages of c. ± 45 a. Figure 1 shows the Th-U ages relative to the year 1950 (BP) and the age depth model. Stalagmite NAH14 was deposited between c. 11,040 and 9520 a BP, indicating that speleothem growth was continuous for c. 1500 years, with an average almost constant growth rate of c. 75 µm per year.

### Proxy results and time series analysis

**Figure 2:**
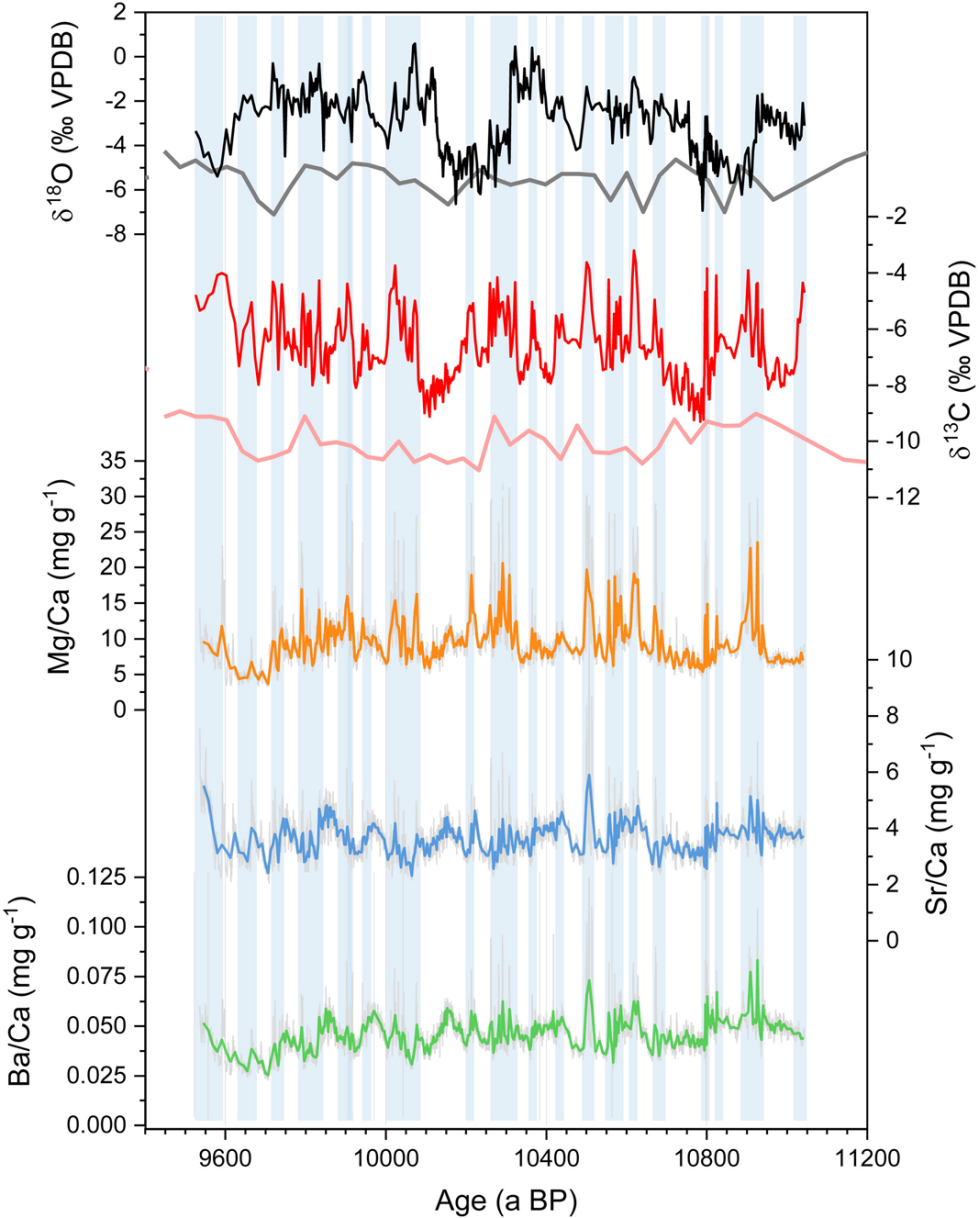
Records of stable isotope and in-situ element/Ca ratios of NAH14. All signals show pronounced variability, but no obvious long-term trend. The stable δ^18^O (black) and δ^13^C (red) isotope records of NAH14 are compared with δ^18^O (gray) and δ^13^C values (light red) from stalagmite CH7 from Chan Hol Cave, also from the Tulúm area of the north-eastern Yucatán peninsula^12^. Light blue vertical bars indicate phases with increased aridity as inferred from the NAH14 δ^13^C and Mg/Ca records.

The stable O and C isotope composition of the stalagmite varies strongly with δ^18^O ranging from − 6 to − 1‰, and δ^13^C ranging from − 10 and − 4‰ (VPDB), respectively. NAH14 has relatively high contents of the elements Mg, Sr, and Ba, with Mg/Ca mass ratios in the range of 5–20 mg g^-1^. Sr/Ca and Ba/Ca ratios show values of 3–6 mg g^-1^ and 0.03–0.07 mg g^-1^, respectively (Fig. 2).

δ^18^O values are uncorrelated with the δ^13^C values (r_δ18O/δ13C_ = 0.26) and minor elemental to Ca ratios. In contrast, the elemental ratios of Sr/Ba and Mg/Ba correlate with r_Sr/Ba_ = 0.79, and r_Ba/Mg_ = 0.64. Even though Mg/Ca shows visible deviation from Sr/Ca the correlation between Sr and Mg remains in a moderate range (r_Sr/Mg_ = 0.52). Also, the δ^13^C values appear visually well correlated with Mg/Ca in a peak-to-peak comparison, which is reflected in a correlation coefficient of r_Mg/δ13C_ = 0.66 for the (down sampled) Mg/Ca times series. All analyzed proxies lack a linear or monotone trend across the period of continuous growth, but exhibit frequent peaks which occur concurrently, where e.g., Mg/Ca values increase by a factor of 2–3. While these short-term excursions towards more positive values occur at approximately decadal scale, the stable C and O isotopes exhibit pronounced swings superimposed on the short-term positive peaks documented above. The stable isotope record has an average temporal resolution of 3–5 years per sample, while the trace element record reaches even sub-annual resolution which allows us to investigate the decadal to centennial periodicities.

**Figure 3:**
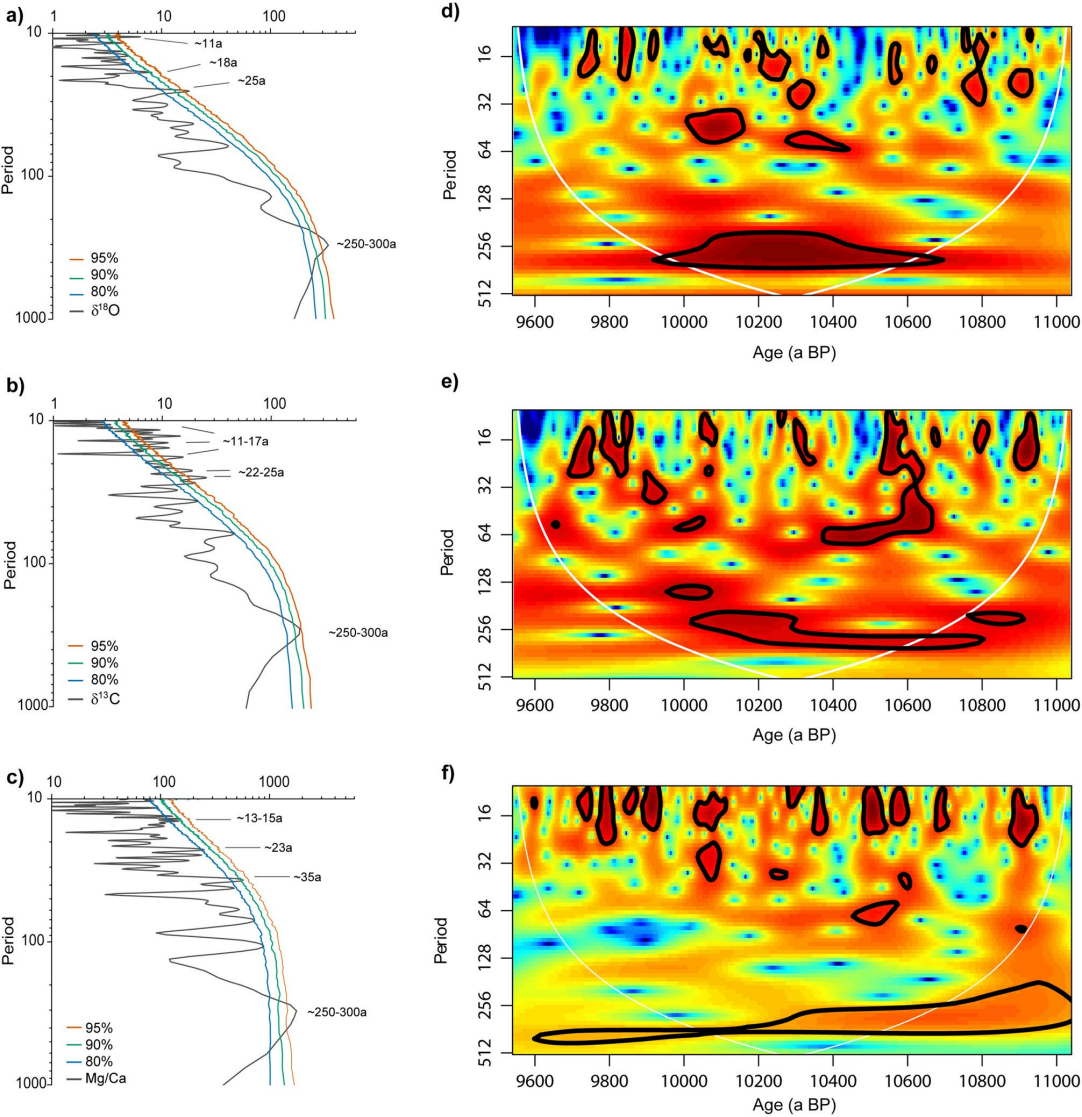
Time series analyses of proxy records: left panels: spectral power diagrams of (**a**) δ^18^O, (**b**) δ^13^C and (**c**) Mg/Ca calculated with REDFIT^39^). Colored lines indicate the calculated AR(1) false-alarm levels of 80% (blue), 90% (green), and 95% (red), respectively. Right panels: continuous wavelet transform spectra computed for the continuous growth period of NAH14 for (**d**) δ^18^O, (**e**) δ^13^C and (**f**) Mg/Ca records down-sampled to 5a-equidistant time series. Red (blue) colors correspond to high (low) values of the transform coefficients (power). Contour lines indicate the 90% significance levels calculated using an AR(1) spectrum with autocorrelation coefficients ρ derived with REDFIT for each proxy record individually (ρ_δ13C_ = 0.75, ρ_δ18O_ = 0.85, ρ_Mg_ = 0.45). The white line indicates the cone of influence where edge effects are not negligible.

Spectral power analyses of δ^13^C, δ^18^O and Mg/Ca confirm the impression from visual inspection and first order correlation analyses (Fig. 3a–c). All three proxies exhibit pronounced power at the centennial scale with periods of c. 250–300 a, and on the multi-decadal and decadal scales. The peaks in the higher frequency range are most pronounced in the δ^13^C record, with significant variability at decadal (c. 11–17 a), and to inter-decadal (22–35a) periods (Fig. 3a). These periods are also pronounced in the Mg/Ca record, even though at lower significance levels (Fig. 3c). The wavelet analysis (Fig. 3d–f) reveals that the decadal to inter-decadal periods are particularly pronounced from c. 10,950 to 10,850, 10,600–10,400 and between 10,100 to 9600 a BP.

**Figure 4:**
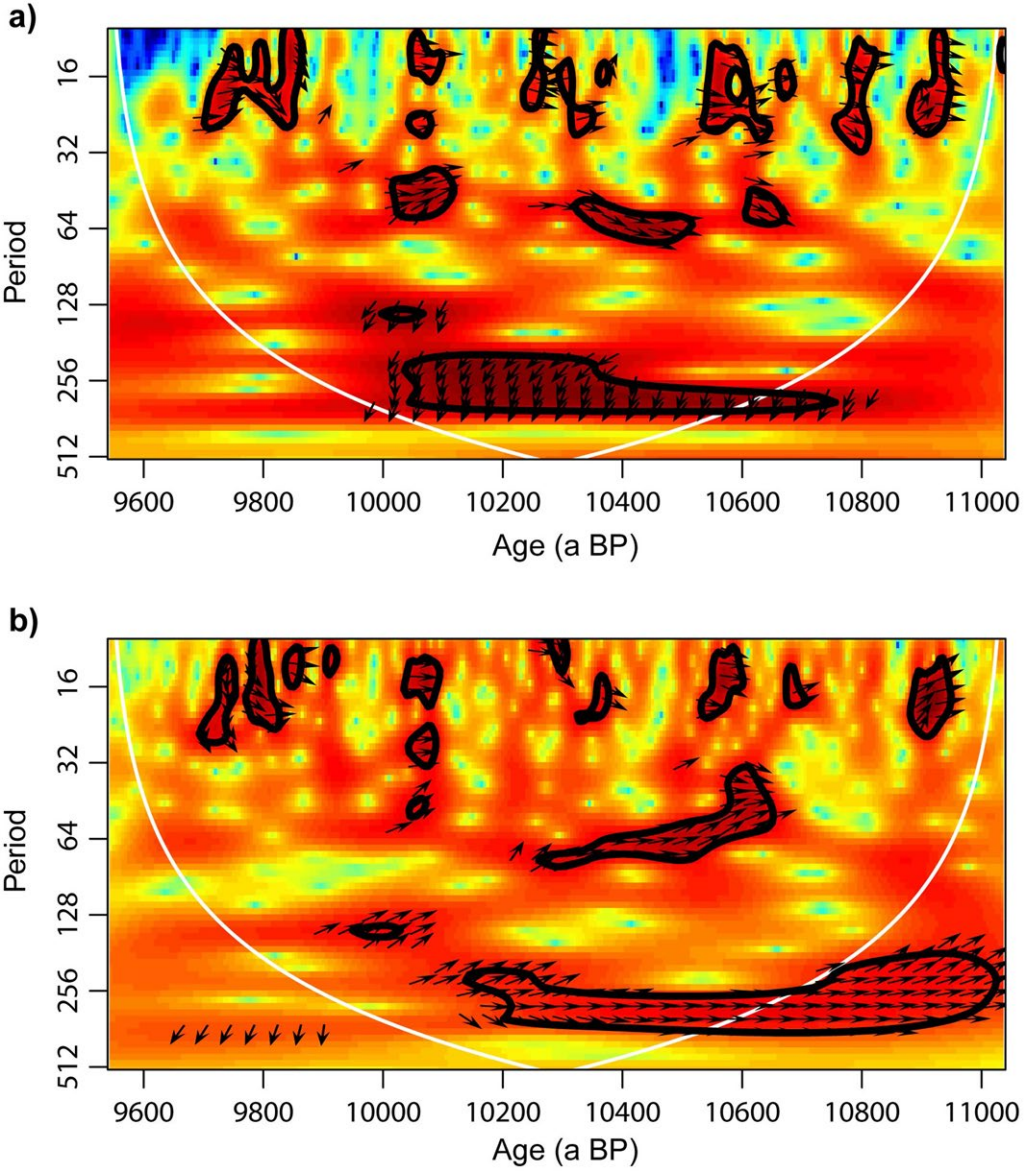
Cross-wavelet transform (XWT) for the (**a**) δ^18^O and δ^13^C record of NAH14, and the (**b**) δ^13^C and Mg/Ca record. Arrows indicate the relative phase between the time series, with arrows pointing towards the right (0°) indicating in-phase variability. Similarly, arrows pointing down (up) (90° (270°)) indicating a lead (lag) of the first time series relative to the second time series. Red (blue) colors indicate high (low) common spectral power. The XWTs show high common power of the δ^13^C record with δ^18^O (**a**) and Mg/Ca (**b**) in the decadal to centennial periods, but δ^13^C values lag the δ^18^O record on the higher periods by c. 100a on the centennial scale.

The observed swings on the centennial scale in the δ^18^O record appear to be out of phase when compared with the NAH14 δ^13^C and Mg/Ca values (Fig. 2). The cross-wavelet transform (XWT) for these proxies unveils high common powers and relative phases in a time–frequency space (Fig. 4), and demonstrates that δ^18^O, δ^13^C and Mg/Ca values are largely in phase on the decadal to multi-decadal scale, whereas the δ^18^O record leads the other time series by about 100 a on the centennial scale.

## Discussion

### The NAH14 hydro-climate record

The apparent co-variability of δ^13^C values and Mg/Ca, Sr/Ca and Ba/Ca ratios is a widespread observation in speleothem geochemical records, and is commonly interpreted as a dominant hydrological control of these proxies^40, 41^. Here, the strongest relationship exists between δ^13^C and Mg/Ca, which are correlated throughout the record (r = 0.66). Prior calcite precipitation (PCP) results in covariations of trace elements such as Sr and Mg, and the evolving δ^13^C composition of the solution, and hence the composition of calcite further down the water flow-line^42, 43^. Thus, higher δ^13^C and Mg/Ca values occur in response to enhanced PCP during periods with lower recharge and allows to interpret synchronous peaks in Mg/Ca and δ^13^C values as a sign of aridity^40, 43^. Sr/Ca and Ba/Ca values in speleothems are often masked by additional processes, such as growth rate or minor detrital contamination, which is reflected by the slightly weaker correlation of these elements to Mg/Ca or δ^13^C values^41, 44^. On the decadal to multi-decadal timescales, frequent excursions towards higher values (i.e. pronounced dry conditions) occur simultaneously not only in δ^13^C and Mg/Ca, but also in the δ^18^O values (Fig. 2). Since the oxygen isotopic composition of the drip water is usually less affected by PCP, this argues for a possible additional kinetic control of the proxies. We suggest that the driving process is most probably carbonate deposition in response to decreasing drip rates and/or enhanced degassing of CO_2_^42, 45^.

Besides the previously mentioned short-term spikes in all proxies, δ^18^O values appear to be largely uncorrelated with δ^13^C or Mg/Ca values and out of phase on centennial timescales (Fig. 4). This is, for example, visible at c. 10,900, c. 10,300, and c. 9600 a BP, when δ^13^C and Mg/Ca values persist on a relatively high level for roughly 100 years longer than the δ^18^O values, while the δ^18^O values switch towards the most negative values of − 4 to − 6 ‰ (Fig. 2). A similar lag between δ^18^O and δ^13^C values is noticeable during the periods of, e.g. 10,800–10,700 and 10,130–10,070 a BP, when δ^18^O values increase, but δ^13^C values remain on a lower level for c. 60–100 a longer.

Following the traditional interpretation of δ^18^O values in tropical speleothems, the very low (high) values could evidence higher (lower) precipitation amount and thus recharge^5, 46–48^, while δ^13^C and Mg/Ca argue for persistently dry (wet) conditions for another century. Thereafter, all three proxies reveal lower (higher) values in concert with the interpretation of increasing (decreasing) precipitation and recharge. This apparent decoupling of the proxies’ responses suggests that the changes in the oxygen isotopic composition of the drip water reflect other effects in addition to precipitation amount and effective recharge, where the latter is regarded as the dominant driver of δ^13^C and Mg/Ca values.

In the low latitudes, enhanced tropical cyclone activity can influence the oxygen isotopic composition of the drip water, due to the particularly low δ^18^O values of tropical cyclone rainfall, and these low δ^18^O values are then transmitted to the growing stalagmite^46, 49^. Intervals in which the NAH14 δ^18^O values drop prior to δ^13^C values may thus indicate decades when the δ^18^O value of the reservoir water in the epikarst—which reflects the recharge-weighted mean of the isotopic composition of precipitation—is no longer able to mask the very negative signature of tropical cyclone rainfall^8, 50^. In the NAH14 record, the uncorrelated shifts towards very negative δ^18^O values occur especially during times with high δ^13^C or Mg/Ca values, which are thought to indicate persistently dry conditions or even multi-annual droughts. This suggests that the previous described effect is most notable when the total water volume of the reservoir is small^46^. The same process might be an explanation for the inverse scenario, when δ^13^C values remain on a lower level, but δ^18^O values increase. This could mean, that the reservoir reached the threshold of sufficient size to be capable to mask the isotopic signature of the tropical cyclone rainfall^46, 49^.

In addition, rainfall δ^18^O in Central America and the Yucatán Peninsula has been shown to be influenced by other effects, such as, e.g., a varying mixture of Caribbean and Pacific moisture sources, which are associated with both a different isotopic composition of the source as well as changing moisture history and trajectories^7, 47^. Moreover, frequent orographic rainfall from trade-winds or northerly cold fronts, may contribute to the isotopic composition of the reservoir water^51^. Thus, we suppose that the speleothem δ^18^O values appear to show a mixed origin related to both precipitation-derived recharge and drought index coupled to regional tropical cyclone activity and varying moisture source and history. Furthermore, kinetic fractionation during the carbonate formation cannot be ruled out completely. Consequently, we interpret the speleothem δ^13^C (and Mg/Ca) values of stalagmite NAH14, which are largely unaffected by moisture source effects, as the more reliable indicators of the underlying structure of local effective recharge, and drought variability.

### Early Holocene precipitation variability on the north-eastern Yucatán peninsula

**Figure 5:**
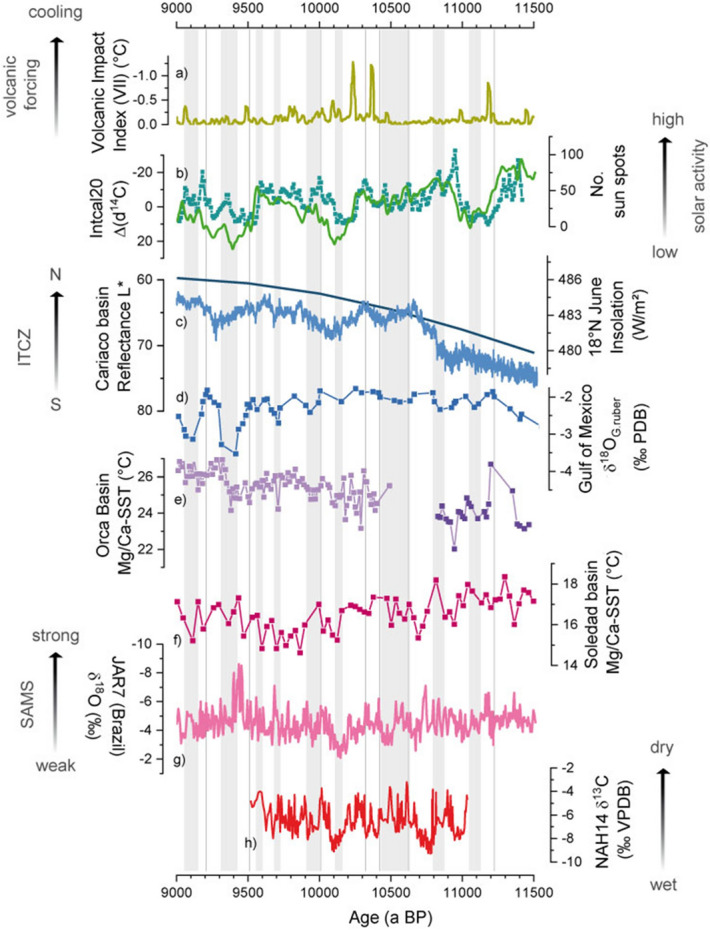
Comparison of the NAH14 δ^13^C record (bottom panel) with early Holocene paleo-environmental records and climate forcing reconstructions. From top to bottom: (**a**) reconstructed volcanic forcing from GISP2^52^, displayed as the Volcanic impact index indicating the potential cooling effect in °C; (**b**) Records of solar activity, as indicated by cosmogenic ^14^C production shown as residual Δd^14^C from Intcal20 (green^53^) and the reconstructed sunspot numbers (cyan^54^) showing the pronounced centennial-scale Suess/de Vries cycle; (**c**) Cariaco Basin Reflectance record^2^ indicating the meridional position of the ITCZ center over northern South America following NH summer insolation (also shown); (**d**) Stacked δ^18^O record planktonic foraminifera G. ruber from seven LOUIS cores^55^ representing the evaporation-precipitation balance in the Gulf of Mexico. According to this record, grey vertical bars indicate the timing and duration of major meltwater floods into the Gulf of Mexico. Vertical black lines indicate additional meltwater pulses from Lake Agassiz as documented by Teller et al.^15^; (**e**) Mg/Ca-based SST from Orca Basin, Gulf of Mexico (light purple:^17^, dark purple:^31^). (**f**) Mg/Ca-based SST reconstruction in planktonic foraminifera from Soledad Basin^28^ demonstrating ENSO-forced tropical Pacific SST variations; (**g**) Speleothem JAR7 δ^18^O values from Jaraguas, Brazil (pink line^33^), reflecting the strength of the South American Monsoon System (SAMS) indicating pan-American precipitation variability compared to the NAH14 δ^13^C values (**h**, red line).

Due to its unprecedented and unique time resolution, the NAH14 record provides the opportunity to investigate decadal to centennial early Holocene climate variability on the north-eastern Yucatán peninsula. In particular, the speleothem covers a 1500 a-long continuous interval between c. 11,040 and 9520 a BP, during a time when previous evidence provides only a fragmentary picture of past precipitation patterns in the tropical Americas as illustrated in Fig. 5 and Fig. S1^18–20^.

Paleo-precipitation records spanning the transition of the last glacial to the mid Holocene in the Caribbean and Central American realm describe a dry-to-wet transition between c. 11,000 and 9000 a BP following the increasing boreal summer insolation, and a relatively stable hydro-climate thereafter^2, 21^ (Fig. 5). In the NAH14 record, however, the above described trend is not identified. In addition, low water tables and terrestrial environments persisted after the deglaciation until c. 8000–9000 a BP in the Lake Chichancanab basin on the northern Yucatán peninsula^4, 11^, and SST reconstructions from the Gulf of Mexico also lack a pronounced orbital trend^17, 31^. This indicates that a progressive northward movement of the mean meridional position of the ITCZ as a whole, as suggested by other proxy records located further to the south^2, 21^, is not (yet) noticeably reflected on the north-eastern part of the Yucatán peninsula (Fig. 5).

δ^18^O, δ^13^C and Mg/Ca values of NAH14 demonstrate that droughts on the decadal to multi-decadal scale were frequently occurring events, as evidenced by repeated peaks towards positive values concurrently in all proxies (Figs. 2 and 3). A higher drought frequency with c. 5–10 drought phases per 100a is identified between 10,950 and 10,800 a, 10,680–10,480 a, 10,330–10,200 a, 10,080–10,000 a, and 9950–9520 a BP. In contrast, during the intervals 10,800–10,680, and 10,200–10,090 a BP, relatively humid and stable conditions prevailed on the north-eastern Yucatán peninsula. In the century between 10,420 and 10,320 a BP, climate was also generally wetter, but humid conditions were interrupted by a drought phase between c. 10,370 and 10,350 a BP. Superimposed on this high-frequency signal, strong swings on the centennial scale indicate pronounced changes in regional rainfall patterns. The NAH14 record suggests that during the more humid phases, decadal-scale droughts were exceedingly rare and climatic conditions were relatively stable. On the other hand, during the already drier intervals, additional, severe droughts occurred much more frequently, with the documented pronounced decadal periodicities of 11–17a in the NAH14 record translating in 5–10 droughts of a duration of up to several years.

Major droughts occurring on a decadal to centennial scale have been widely reported for Mesoamerica during the last millennia^4–6, 24^, thus reflecting a pattern similar to that preserved in NAH14 for the early Holocene. Increased drought frequency during the late Holocene, however, came along with generally drier conditions, resulting from a progressive southward displacement of the ITCZ after 7000–3000 a BP^11, 21, 30^. In contrast, the mid-Holocene northernmost position of the ITCZ was accompanied by reduced precipitation variability. Similarly, the here observed early Holocene decrease in precipitation variability occurs during presumably more humid phases, which is indicative of an extension of the ITCZ on these timescales. An expansion of the convective centers positions the Yucatán peninsula within the seasonal latitudinal bounds of the ITCZ for a longer period each year^30, 56^. In contrast, during the drier phases of the record, the study site experienced prolonged dry seasons, which leads to a higher seasonal contrast, and thus higher precipitation variability.

In addition to the absent, previously discussed long-term orbital trend, the centennial- to millennial scale swings between wet and dry conditions in the north-eastern Yucatán peninsula appear to be largely unrelated to the high-resolution reflectance record from the Cariaco basin (Fig. 5), which has been associated with the latitudinal position of the ITCZ^2^. The precipitation at our locality is likely sensitive to changes in the northernmost margin of the ITCZ, responding to both meridional ITCZ displacement but also to expansion and contraction of the band of seasonal ITCZ movement (Fig. 1)^3, 57^. This discrepancy thus hints towards a more complex behavior of the ITCZ and its northernmost extent over Mesoamerica.

### Forcing mechanisms of precipitation variability

On seasonal and inter-annual timescales, the distribution of precipitation over the Yucatán peninsula is modulated by the so-called Pacific-Atlantic ‘inter-basin mode and is thus influenced by several forcing mechanisms, such as ENSO activity, radiative forcing, or variations in Atlantic SSTs^4, 6–8, 22, 23^. Deglacial and early Holocene records from the tropical Atlantic realm suggest a link of local precipitation to sub-centennial to millennial freshwater input into the North Atlantic and the Gulf of Mexico^19, 20^. The comparison of NAH14 speleothem data with marine records from the Gulf of Mexico (Fig. 5), shows that meltwater flux into the Gulf of Mexico resulted in decreasing SSTs and drier conditions on the north-eastern Yucatán peninsula^17, 31, 55^. The centuries-long dry–wet cycles also roughly agree with previous paleoclimatic evidence deduced from lacustrine sediments of Lake Petén Itza, southern Yucatán, suggesting a series of wet–dry cycles concurrent to pre-boreal meltwater flooding events in the Gulf of Mexico^15, 19, 55^.

In addition to these single events, spectral analyses revealed dominant forcing mechanisms with pronounced periodicities of 11–17 a, 25–35 a and c. 250 a (Fig. 3). The strong spectral power of these periods reflect reported periodicities in records of modern Mesoamerican climate variability, and are very similar to solar activity cycles (11 a Schwabe solar (or sunspot) cycle and reoccurrence of the c. 210–220 a de Vries/Suess solar cycle^58, 59^. A strong link between the NAH14 proxies and solar forcing is further supported by the agreement with a reconstruction of sunspot numbers^54^, as well as solar activity reflected in the residual cosmogenic ^14^C^53^, as shown in Fig. 5. A relation of early Holocene speleothem δ^18^O values with solar activity has been previously suggested by Stinnesbeck et al.^12^ to better constrain the chronology of a speleothem record from Chan Hol cave on the north-eastern Yucatán peninsula between 9000 and 13,000 a BP. We here confirm this link, but based on a precisely dated record of much higher temporal resolution.

The influence of solar variability on tropical hydro-climate has been predicted by model studies^60, 61^, and especially the centennial-scale Suess-cycle is documented in various early to late Holocene records throughout the tropical and subtropical Americas^4, 12, 18, 24, 34, 56, 62^. In particular, solar cycles modulate ENSO variability via the so-called “ocean dynamical thermostat” response, because negative (positive) radiative forcing results in a dynamic warming (cooling) of the eastern tropical Pacific^26, 28, 60, 63^. ENSO itself influences the precipitation pattern over the Yucatán peninsula, where a warm El Niño event is associated with drier conditions^6, 23^. As a consequence, the question of the role of solar activity in climate variability of the tropical Americas has been frequently debated^7, 24, 62^. We here present a record that is sufficiently well resolved and clearly provides evidence for an empirical similarity between solar activity and early Holocene Mesoamerican precipitation. The origin of this coupling, however, may be suspected in ENSO activity, which evolved over the course of the Holocene^26, 27, 64^. During the early to mid-Holocene, a dampening of ENSO activity has been reported^29^, while other studies suggest that ENSO variance was close to modern levels^65^. For example, an early Holocene (c. 9500 a) model experiment suggests that freshwater flux and remnant ice sheet forcing factors led to significant remote responses in the tropical Pacific strengthening the amplitude of eastern Pacific El Niño events^64^. The visual inverse relationship of the NAH14 record to Pacific SSTs^28^ provides additional support for a strong influence of tropical Pacific dynamics on Mesoamerican precipitation (Fig. 5). The late Holocene onset of major dry events after c. 3000–4000 a BP coincided with ENSO and ITCZ changes, with an increasing dominance of the Pacific Ocean^18, 56^. Consequently, the persistent decadal to centennial solar cycle forcing of the regional hydro-climate, appears to be modulated by an ENSO activity which seems to be similar as the one in late Holocene leading to pronounced decadal droughts in northern Yucatán and Mesoamerica.

The spectral analyses also suggest the presence of inter-decadal periods of c. 25–35 years in the NAH14 record (Fig. 3). Long-term ENSO variability may modulate the Pacific Decadal oscillation (PDO), which is not a single physical mode of ocean variability, but rather the sum of several processes with different dynamic origins^66^. Enhanced rainfall over Mesoamerica on the multi-decadal scales may be also related to a warmer North Atlantic Ocean related to a pattern similar to the late Holocene Atlantic multidecadal oscillation (AMO)^6, 23^. However, the wavelet analyses of the NAH14 record show that these multi-decadal periods are not a persistent feature in this record and only significant during a few, short intervals. This is similar to the results of Lachniet et al.^7^, who reported no clear link to the PDO or AMO regarding the strength of wet season rainfall in the Mesoamerican monsoon based on late Holocene stalagmite records from south-western Mexico. Similarly, recent studies suggest that especially the AMO is no internal oscillation of Earth’s climate, but rather an externally forced quasiperiodic variation^67^. In particular, recent research suggested an influence of volcanic eruptions and aerosol emission on the regional evaporation/precipitation balance in Mesoamerica, and that these exacerbated or prolonged multi-decadal drought intervals during the past centuries in Mesoamerica^8, 67^. In Fig. 5, we compare our data with a reconstruction of volcanic forcing generated from sulphate concentrations of the Greenland GISP2 ice core^52^. This comparison shows, that the number of recorded droughts in the NAH14 proxies exceeds by far the number of known strong volcanic eruptions. Coeval to two major volcanic events at c. 10,400 and 10,250 a BP apparently strong dry events are recorded in the NAH14 proxies. In contrast, the most pronounced dry phases, such as for example after c. 10,930 a, 10,650 a, or 10,050 a, rather occur in absence of major detected volcanic eruptions in the ice core sulphate loads. Even though, we cannot exclude the possibility that volcanic forcing might have punctually contributed to or exacerbated droughts occurring during the early Holocene, we argue that other mechanisms were probably more dominant.

A large-scale comparison of the NAH14 record with other speleothem-based reconstructions from North and South America highlights the associated link of both low and mid-latitude precipitation patterns during the early Holocene. The centennial-scale dry/wet cycles on the north-eastern YP are largely anti-phased to precipitation recorded in speleothems from Brazil^33^ (Fig. 5) and Peru^32^ (Fig. S1). Both records reflect the strength of the South American Monsoon System (SAMS), thus the visual anti-correlation is interpreted as an expression of a hemispheric anti-phasing in the low-latitudinal Americas, similar to late Holocene observations^9^. In addition, an inverse relationship of precipitation in the northern American low- and mid-latitudes may be suggested by comparing the NAH14 record with the hydro-climate reconstructions from the Great Basin^35^ and New Mexico^34^ (Fig. S1). Despite the lower temporal resolution of these records compared to NAH14, periods of higher precipitation in Central America appear to coincide with drier conditions in New Mexico, and more northerly moisture sources in the Great Basin. This comparison thus further supports previous evidence that the meridional displacement or expansion and contraction of the ITCZ shifts the Hadley cell and drives circulation processes in the higher latitudes^9, 34^.

Southward shifts of the ITCZ and the Hadley Cells reduce the meridional pressure gradient in the northern hemisphere, inducing expansion and southward displacement of the polar and mid-latitude pressure cells^9^. Consequently, the polar jet and westerlies (Fig. 1) shifted southward and weakened, and monsoonal systems propagated less far northward^1^. Similar to the past 2000 a, the observed low-latitude hemispheric anti-phasing over decadal-centennial timescales in response to a superposition of meridional shifts and expansions/contractions of the ITCZ during the early Holocene, is most likely a result of a combination of external forcing. These encompass in particular solar activity and maybe also volcanic eruptions, but also internal feedback mechanisms, including meltwater fluxes into the Gulf of Mexico and the North Atlantic Ocean.

## Conclusions

The high-resolution precipitation record presented here from the north-eastern Yucatán peninsula provides a valuable, unprecedented snapshot of early Holocene climate variability for the area. In particular, our research suggests a strong decadal‐ to centennial‐scale climatic and hydrologic variability during the early Holocene, a time without anthropogenic radiative aerosol forcing. This study further demonstrates that the reconstruction of sub-decadal drought occurrence is feasible on the north-eastern Yucatán peninsula.

The elemental (Mg/Ca) and isotopic (δ^13^C) speleothem records provide evidence of drought frequency dominantly modulated by solar forcing on both the c. 11a sunspot cycle, as well as the c. 250 a de Vries/Suess periods of solar activity as estimated from residual Δd^14^C. In absence of a long-term trend related to insolation or the retreat of the Laurentide ice sheet, the precipitation variability on the north-eastern Yucatán peninsula during the interval from 11,040 and 9520 a BP was characterized by pronounced centennial variability. These swings were likely related to a combination of solar forcing and additional impacts by freshwater flux into the Gulf of Mexico and the North Atlantic, influencing both local evaporation/precipitation ratios and the position of the ITCZ.

Despite the global atmospheric reorganizations during the early Holocene, the frequently recurring drought events on the decadal scale suggest that one of the leading modes of precipitation variability during the late Holocene, the El Nino-Southern Oscillation, was also active during the time of our record. In contrast, the partial absence of multi-decadal modes in our record questions the existence of a persistent internal oscillation of the climate system on these scales. Our analyses suggest an overall similar precipitation pattern compared to the late Holocene, while the climate boundary conditions have been very different. Thus, we suggest that in absence of strong volcanic or anthropogenic aerosol forcing, our study underscores the previously suggested link of tropical hydro-climate with a strong ENSO activity driven by solar forcing of the tropical Pacific.

The coherence of our data with other low- and mid-latitude speleothem records from the Americas indicates that low- to mid-latitude teleconnections persisted on the centennial timescales during the early Holocene. We argue that the observed low-latitude see-saw on centennial timescales shows that the same forcing mechanisms and teleconnections dominated the hydrological regime in the tropical Americas during the early and late Holocene, and that solar forcing may explain much of the observed variability throughout the Americas.

## Methods

### Analytical methods

For this study, the growth history of NAH14 has been investigated by high-precision ^230^Th-U dating using a multi-collector inductively coupled plasma source mass spectrometer (MC-ICPMS, Thermo Fisher Neptune^plus^). Sub-samples of 80–130 mg were cut using a diamond wired band saw along the major growth axis. Chemical preparation of the samples and analytical methods at the Institute of Environmental Physics, Heidelberg University, followed previously reported procedures^44^. The calculations of activity ratios and ^230^Th-U ages were performed using the half-lives from^68^. Age uncertainties are quoted at the 2σ-level and do not include half-life uncertainties. All ages are reported relative to the year 1950 (BP). The age-depth model for the stalagmite proxies was constructed using the algorithm ‘COPRA’^36^.

Trace element analysis was performed via laser ablation ICP-MS using a 193 nm ArF excimer laser (NWR193^UC^ by New Wave Research) coupled to an inductively coupled plasma quadrupole mass spectrometer (Thermo Fisher iCAP-Q) at the Institute of Environmental Physics, Heidelberg University. Line scans were performed at a speed of 20 µm/s along the growth axis of NAH14 using a rectangular spot size of 15 × 150 µm (with the longer side being vertical perpendicular to the track) and a scan speed of 20 µm/s. To avoid potential surface contamination, the scan path was pre-ablated at a scan speed of 80 µm/s. The repetition rate of the laser pulses was 20 Hz, and each isotope was measured every 100 ms on the mass spectrometer. Background counts were measured with the laser in off mode and subtracted from the raw data. In order to account for matrix effects, the blank corrected count rates of the analyzed isotopes (^24^Mg, ^27^Al, ^31^P, ^34^S, ^88^Sr, ^138^Ba, ^232^Th, ^238^U) are calculated relative to the intensity of the ^44^Ca signal of the internal standard element Ca. The silicate glass NIST SRM 612 was analyzed for external calibration of the trace element analyses using the reference values by Jochum et al.^69^. The resulting element to Ca ratios are presented as mass concentration ratios.

Samples for stable isotope analysis were drilled directly adjacent to the trace element track at a spatial resolution of 250 µm using a micro mill. All samples were analyzed using an isotope ratio mass spectrometer (IRMS) (Thermo Scientific Delta V coupled with a Gasbench II at Elemtex Ltd., Cornwall, UK). Quality control is performed using a Carrara marble standard (δ^13^C = 2.10‰, δ^18^O = − 2.01‰) and a second in-house calcite standard (δ^13^C = 2.89‰, δ^18^O = − 6.15‰), calibrated directly against NBS18 and NBS19. The reported long-term precision (1sd) is 0.022‰ for δ^13^C and 0.040‰ for δ^18^O values. All δ-values are reported relative to Vienna Pee Dee Belemnite (VPDB) standard.

### Correlation and time series analysis

To quantify the nature of the observed variability on the different timescales in the proxy record we performed dedicated time serial analytical methods, including correlation, spectral and wavelet analyses. Correlation analysis was performed with a test statistic based on Pearson’s product moment correlation coefficient r (x, y) following a t-distribution with length(x)-2 degrees of freedom. Reported correlation coefficients are all significant at the 0.05 level. For the calculation of the correlation coefficients, element/Ca ratios were interpolated to the lower resolution of the stable isotope records. Spectral analysis was conducted using the algorithm “REDFIT”^39^, as implemented in the open source software R package “dplR”^70^. REDFIT fits a first-order autoregressive (AR1) process directly to unevenly spaced time series. Wavelet analyses were performed using the R package “Biwavelet”^71^.

## Supplementary Information

Supplementary Information.

## Data Availability

Data associated with this study will be uploaded to the open data library PANGAEA (www.pangaea.de) and will be available after acceptance of this paper.
