# Supplementary material for: Solar forcing of early Holocene droughts on the Yucatán peninsula
Source: Sci Rep. 2021 Jul 6;11:13885. doi: 10.1038/s41598-021-93417-z (PMC8260582; doi:10.1038/s41598-021-93417-z)
Supplement: Supplementary file 1 — Supplementary Information. [file 41598_2021_93417_MOESM1_ESM.pdf]

Supplementary material to

Solar forcing of early Holocene droughts on the Yucatán peninsula

Sophie F. Warken[1,2*], Nils Schorndorf[1,2], Wolfgang Stinnesbeck[2], Dominik Hennhoefer[3], Sarah R. Stinnesbeck[4], Julius Förstel[1], Simon D. Steidle[1,5], Jerónimo Avilés Olguin[6], Norbert Frank[1]

[1] Institute of Environmental Physics, Heidelberg, Germany
[2] Institute of Earth Sciences, Heidelberg, Germany
[3] Department of Earth Sciences, Khalifa University, Abu Dhabi
[4] Institute of Earth Sciences, Natural History Museum, Karlsruhe, Germany
[5] Institute of Geology, University of Innsbruck, Innsbruck, Austria
[6] Museo del Desierto, Saltillo, Coahuila, Mexico

*correspondence to: Sophie Warken, swarken@iup.uni-heidelberg.de

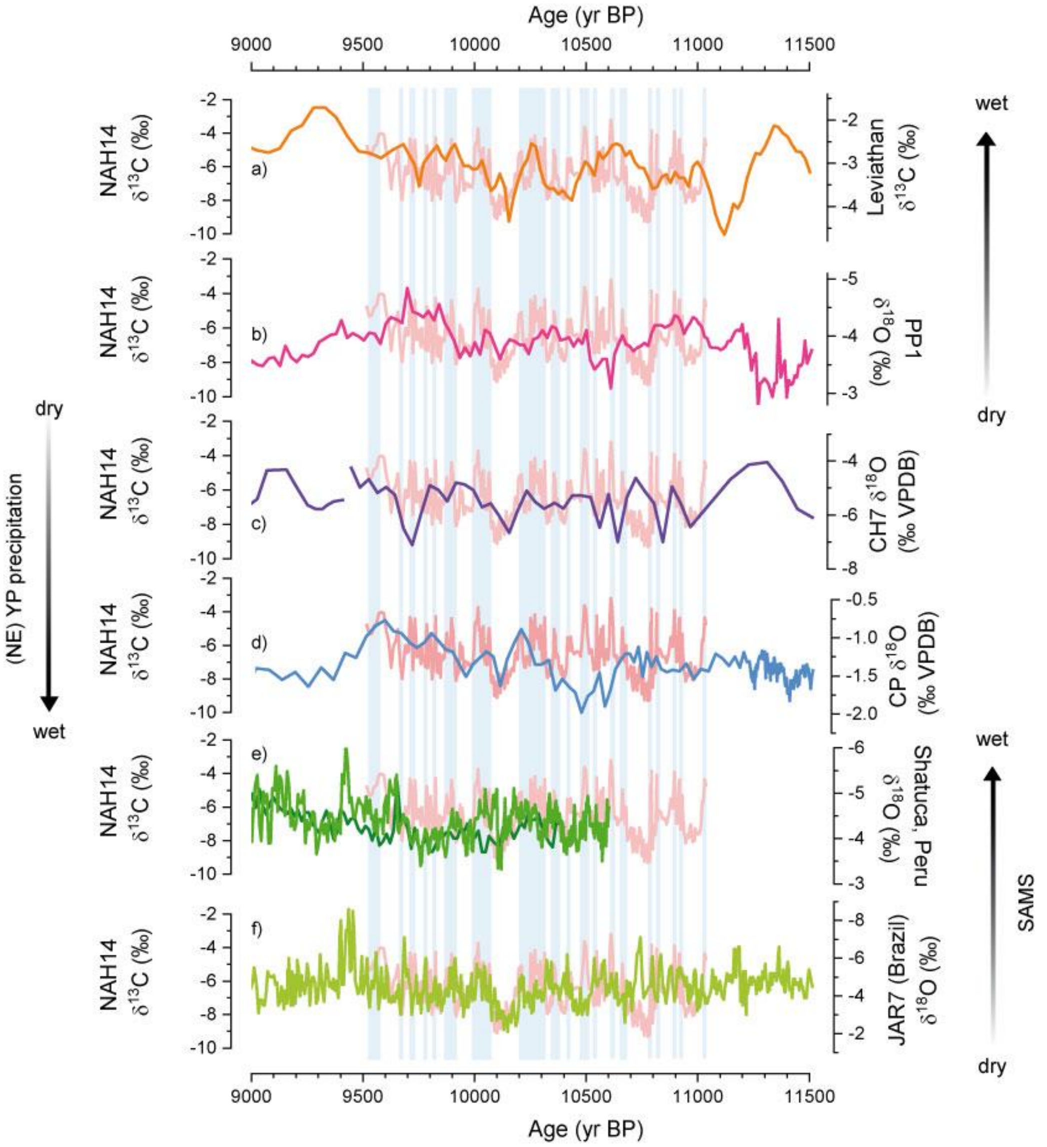

Figure S 1: Large-scale comparison of the NAH14 speleothem $\delta^{13}C$ record (red line) with other early Holocene speleothem hydro-climate records from North, Central and South America. From top to bottom: (a) Leviathan Cave $\delta^{13}C$ record ([orange line, 1]); (b) Stalagmite PP10 $\delta^{18}O$ record from Pink Panther Cave, New Mexico [pink line, 2]. Both records from North America reflect wet/dry conditions in response to the strength of the northern hemispheric westerlies; (c) Speleothem CH7 $\delta^{18}O$ values (purple line [3]) and (d) CP $\delta^{18}O$ values (Cuba, light blue line [4]), indicating low-resolution precipitation variability on the NE Yucatán peninsula and western Cuba; (e) Stalagmite Sha2 and Sha3 $\delta^{18}O$ records from Shatuca, Peru [green line, 5], and (e) JAR7 $\delta^{18}O$ values from Jaraguas, Brazil [light green line, 6], both reflecting the strength of the SAMS. The light red curves in each panel show the NAH14 speleothem $\delta^{13}C$ record to allow a direct comparison to the other records. Vertical light blue bars indicate dry spells on the NE Yucatán peninsula, as indicated from the NAH14 record (this study).

Table S 1: ^{230}Th/U ages and activity ratios measured in stalagmite NAH14. Activity ratios and ages are calculated using the decay constants by Cheng et al., 2013[7]. Ages were corrected for initial Th with an initial (^{230}Th/^{232}Th) activity ratio of 3.5 ± 1.75, and assuming secular equilibrium of the detritus. Corrected ages are given relative to the year 1950 (BP). The outlying age at 98.5mm dft (marked with a *) is not considered for the age model due to a likely contamination with old carbonaceous material, e.g. from the encrusted flowstone visible in Figure 1.

**Sample ID**	**^{238}U**	**Error**	**^{232}Th**	**Error**	**(^{230}Th/ ^{238}U)**	**Error**	**(^{230}Th/ ^{232}Th)**	**Error**	**δ^{234}U**	**Error**	**$\delta^{234}U_{ini}$**	**Error**	**Age (uncorr.)**	**Error**	**Age (corr.)**	**Error**	**dft**
	**(ng/g)**	**(abs.)**	**(ng/g)**	**(abs.)**	**(Act. ratio)**	**(abs.)**	**(Act. ratio)**	**(abs.)**	**(‰)**	**(‰)**	**(‰)**	**(‰)**	**(ka)**	**(ka)**	**(ka BP)**	**(ka)**	**(mm)**
NAH14-1mm	972.695	0.069	0.16404	0.00033	0.08630	0.00031	1572.4	6.5	18.8	1.0	19.3	1.0	9.649	0.038	9.559	0.039	1
NAH14-5,5mm	745.243	0.044	0.06070	0.00020	0.08766	0.00047	3312	21	19.59	0.92	20.14	0.95	9.800	0.057	9.722	0.056	5.5
NAH14-16,5mm	1226.457	0.054	0.05149	0.00016	0.08844	0.00030	6457	30	14.78	0.78	15.20	0.80	9.941	0.036	9.866	0.036	16.5
NAH14-23mm	1164.147	0.059	0.08125	0.00018	0.08929	0.00031	3915	16	16.00	0.74	16.46	0.76	10.029	0.038	9.950	0.038	23
NAH14-40.5mm	866.894	0.040	0.05160	0.00012	0.09091	0.00035	4682	21	16.49	0.93	16.97	0.95	10.214	0.043	10.138	0.042	33.5
NAH14-40,5mm	1348.853	0.063	0.08046	0.00017	0.09131	0.00028	4680	17	18.89	0.71	19.44	0.73	10.236	0.034	10.158	0.034	40.5
NAH14-50,5mm	1166.236	0.061	0.07419	0.00017	0.09283	0.00034	4462	19	19.21	0.54	19.78	0.55	10.410	0.041	10.333	0.040	50.5
NAH-14-59mm	1334.848	0.071	0.05719	0.00024	0.09354	0.00045	6663	42	17.8	1.0	18.3	1.0	10.510	0.054	10.435	0.054	59
NAH14-61.5mm	908.340	0.053	0.36382	0.00073	0.09479	0.00052	725.9	4.2	16.88	0.94	17.39	0.97	10.667	0.063	10.548	0.067	61.5
NAH14-63mm	1113.827	0.066	0.02744	0.00008	0.09448	0.00042	11746	62	17.3	1.0	17.8	1.1	10.626	0.052	10.553	0.051	63
NAH14-68mm	1280.486	0.066	0.04521	0.00009	0.09480	0.00024	8208	27	17.94	0.49	18.49	0.51	10.656	0.029	10.582	0.029	68
NAH14-77mm	1003.236	0.050	0.06200	0.00015	0.09654	0.00041	4796	23	16.34	0.96	16.85	0.99	10.881	0.049	10.804	0.049	77
NAH14-82,5mm	1337.418	0.065	0.07381	0.00017	0.09671	0.00033	5346	22	19.25	0.64	19.84	0.66	10.868	0.040	10.791	0.039	82.5
NAH14-91mm	1472.448	0.077	0.05552	0.00015	0.09654	0.00032	7819	34	16.92	0.64	17.44	0.66	10.874	0.039	10.799	0.039	91
NAH14-94mm	1217.573	0.057	0.09532	0.00026	0.09751	0.00036	3809	18	15.34	0.75	15.82	0.78	11.008	0.044	10.928	0.044	94
NAH14-97mm	1340.093	0.061	0.08148	0.00026	0.09773	0.00034	4915	23	16.23	0.66	16.74	0.68	11.023	0.041	10.946	0.041	97
NAH14-98,5mm*	1384.649	0.105	0.06287	0.00026	0.10325	0.00043	6985	41	18.32	0.66	18.93	0.68	11.654	0.052	11.580	0.052	98.5
NAH14-103mm	1602.210	0.100	0.04614	0.00019	0.09804	0.00039	10370	60	16.48	0.61	17.00	0.63	11.056	0.048	10.982	0.047	103

References

1. Lachniet, M.S., et al., *Great Basin Paleoclimate and Aridity Linked to Arctic Warming and Tropical Pacific Sea Surface Temperatures*. Paleoceanography and Paleoclimatology, 2020. **35**(7): p. e2019PA003785.
2. Asmerom, Y., et al., *Solar forcing of Holocene climate: New insights from a speleothem record, southwestern United States*. Geology, 2007. **35**(1).
3. Stinnesbeck, W., et al., *The earliest settlers of Mesoamerica date back to the late Pleistocene.* PLoS One, 2017. **12**(8): p. e0183345.
4. Fensterer, C., et al., *Millennial-scale climate variability during the last 12.5 ka recorded in a Caribbean speleothem.* Earth and Planetary Science Letters, 2013. **361**: p. 143-151.
5. Bustamante, M.G., et al., *Holocene changes in monsoon precipitation in the Andes of NE Peru based on δ18O speleothem records.* Quaternary Science Reviews, 2016. **146**: p. 274-287.
6. Novello, V.F., et al., *A high-resolution history of the South American Monsoon from Last Glacial Maximum to the Holocene.* Scientific Reports, 2017. **7**(1): p. 44267.
7. Cheng, H., et al., *Improvements in 230Th dating, 230Th and 234U half-life values, and U--Th isotopic measurements by multi-collector inductively coupled plasma mass spectrometry*. Earth and Planetary Science Letters, 2013. **371**: p. 82-91.
